# Supplementary material for: A novel enrichment strategy reveals unprecedented number of novel transcription start sites at single base resolution in a model prokaryote and the gut microbiome
Source: BMC Genomics. 2016 Mar 8;17:199. doi: 10.1186/s12864-016-2539-z (PMC4782308; doi:10.1186/s12864-016-2539-z)
Supplement: Additional file 2: — contains Table S1. (ZIP 9431 kb) [file 12864_2016_2539_MOESM2_ESM.zip › Supplemental text.docx]

**Supplement**

**Note A**

**Capping 5’ triphosphorylated RNA with modified GTP:**

Vaccinia Capping Enzyme (VCE) is known to cap RNA using GTP at either a 5' triphosphate or 5' diphosphate terminus with similar efficiency while VCE is ineffective when 5' monophosphate RNA is used[1, 2]. We assessed the ability of VCE to cap a 5’ triphosphorylated RNA with modified GTP. VCE and a T7 RNA transcript were incubated with either 3’ desthiobiotin-TEG-GTP (DTB-GTP) or GTP (Supplemental Fig. 1). The GTP reaction converted the 25 mer 5' triphosphorylated transcript to a slower moving electrophoretic band due to the transfer of GMP from GTP to the 5' end of the acceptor RNA. Furthermore, VCE converted the transcript to slower moving band when DTB-GTP was used in place of GTP. Using a similar assay, we also determined that 5' diphosphate RNA (Supplemental Fig. 2) was also an acceptor for DTB-GTP. The position of the biotinylated linker on the sugar is critical, as a 2' DTB-GTP does not act as a cap donor (Supplemental Fig. 2 and Supplemental Fig. 3). Next we demonstrate that capping of a 5' monophosphate RNA with DTB-GTP does not occur at a detectable level (Supplemental Fig. 4). Taken together these results indicate that VCE displays similar enzymatic properties when the GTP is replaced with 3' DTB-GTP . Our data further shows that VCE is specific for 5’ di and triphosphorylated RNA characteristic of prokaryotic primary transcripts over 5’ monophosphorylated RNA characteristic of degraded and processed transcripts. To achieve effective enrichment, the DTB-capped RNA must be specifically captured on streptavidin magnetic beads and eluted with biotin. To assess this, we bound, a uniformly ^32^P-labeled 300mer T7 transcript that had been incubated with either 3' DTB-GTP, 2' DTB-GTP or GTP and VCE. The resulting RNAs were adsorbed to streptavidin magnetic beads washed and eluted with biotin and the radioactivity from the eluted fractions was measured. The results show that the transcript that was reacted with 3’ desthiobiotin-TEG GTP was selectively bound to the streptavidin beads and eluted with biotin whereas when 2’ DTB-GTP or GTP was used there was little or no recovery of the transcript (Supplemental Fig. 3).

**Decapping DTB-GTP capped RNA:**

The strategy here for the preparation of sequencing libraries is based on ligation of the 5’ end of transcripts and therefore requires a 5’ monophosphorylated RNA. Thus, the modified cap needs to be removed leaving a free 5’ monophosphorylated end. In accordance with a previous study [3], we demonstrate by analysis on Urea-PAGE that RNA 5’ pyrophosphohydrolase (RppH) is capable of removing a 7mG cap, as well as a 3' desthiobiotin-G cap from RNA (Supplemental Fig. 5 and Supplemental Fig. 6). In summary, Vaccinia Capping enzyme (VCE) effectively caps a triphosphorylated 5’ RNA end with 3’ DTB-GTP and RppH effectively decaps DTB-GTP-capped RNA.

**Note B**

**Cappable-seq demonstrates specificity for TSS:**

The accurate description of TSS in prokaryotes relies on the differentiation of the 5'-triphosphorylated end characteristic of primary transcripts from the 5’ monophosphorylated end characteristic of processed sites. Therefore it is key for any method to distinguish primary from processed transcripts.

The majority of published bacterial TSS are determined by treating RNA samples with a 5’ to 3’ exonuclease from *S. cerevisiae*, Xrn1 also known as Terminator™ exonuclease. This exonuclease preferentially degrades RNAs containing a 5’ monophosphate, therefore resulting in an apparent enrichment of primary transcripts containing 5'-triphosphates. This method is generally referred to as TEX. A Xrn1 treated library is often compared with a non-enriched control library consisting of non-Xrn1 treated RNA to remove false positives. The resulting method is referred to as differential RNA-seq or dRNA-seq. We compared Cappable-seq to published dRNA seq dataset of *E. coli* MG1655 grown in minimal media [4] to evaluate the performance of both methods in defining TSS.

To this end, we analyzed the levels of the ribosomal RNAs (rRNA) and transfer RNA (tRNA) as

a proxy to evaluate the fate of processed transcripts in Cappable-seq and dRNA-seq libraries.

Ribosomal (rRNA) and transfer RNA (tRNA) are well characterized representatives of processed RNA generated from a precise endoribonuclease cleavage of the primary transcripts [5–7] and consequently, the 5’ends of mature tRNAs and rRNAs are monophosphates. We found that the relative amount of tRNA and rRNA dropped from an overwhelming majority of 86% in our non-enriched control library to only 4 % in the Cappable-seq library (Supplemental Fig. 7A). The remaining 4 % of tRNA and rRNA in Cappable-seq maybe a combination of contaminating tRNA and rRNA or bona fide TSS within ribosomal genes (see text below). These results shows Cappable-seq does not capture processed RNA. The same analysis performed using published dRNA-seq data [4] reveals ribosomal and tRNA content in fact increased from 35% in the control library to 56 % after Xrn1 treatment (Supplemental Fig. 7A). These results show that Xrn1 treatment does not remove the majority of reads mapping to tRNA and rRNA and thus, does not appear to completely degrade the processed ribosomal RNA. This observation is in sharp contradiction with the expected activity of the enzyme but in agreement with previous reports of TEX being blocked by secondary structure [8–10].

To confirm this result, we further investigated this apparent contradiction by analyzing the annotated processed 5’ ends at single base resolution for both the rRNAs and tRNAs and found only a small subset of tRNA and rRNA processed sites are depleted after Xrn1 treatment (Supplemental Fig. 7B). More specifically, rRNA processed sites that are enriched in dRNA-seq correspond to the 5S rRNAs. Most of the tRNAs are enriched in dRNA-seq except for the methionyl-tRNAs (MetU, MetT, MetZ, MetW, MetV and MetY) that are significantly depleted.

In contrast all of the rRNAs and all but one of the 82 tRNAs’ processed sites are strongly depleted with Cappable-seq (Supplemental Fig. 7B). Taken together, these results demonstrate that Xrn1 has a differential activity amongst 5’ monophosphorylated RNA substrates leaving intact a number of 5’ monophosphate processed ends. In the case of the known processed sites that we have looked at, the distinction between TSS and processed sites cannot be made when using Xrn1. In fact Thomason et al. [4] annotated 40 of these tRNA and rRNA processed sites as TSS.

We hypothesized that the inability of Xrn1 to degrade certain processed transcripts can be generalized beyond the well-characterized processed sites. To this end, we calculated the enrichment score for all positions in the genome for both Cappable-seq and dRNA-seq data. Cappable-seq separates the RNA into two distinct enriched and depleted populations. We mapped a collection of TSS from regulonDB [11] to this data and found the enriched population contains most of the known TSS (Supplemental Fig. 7C). This result suggests that the enriched population represents TSS and the depleted population represent processed sites. While dRNA-seq also separates into two distinct populations, both distributions are overlapping to a greater extent than Cappable-seq. As the difference of the enrichment score between enriched and depleted is greater for Cappable-seq than dRNA-seq, Cappable-seq more clearly distinguishes between the two populations, demonstrating that Cappable-seq has a higher specificity than dRNA-seq for triphosphorylated ends.

We individually analyzed the TSS from dRNA-seq [4] that are depleted in Cappable-seq. In addition to the known processed 5’ ends of tRNA and rRNA annotated as TSS with dRNA-seq, we found intragenic sites within rRNA genes, the processed site of the transfer-messenger RNA (tmRNA) and a position in the intragenic region of the SerA gene. These results suggest that the Xrn1 reaction does not go to completion. In fact, others have reported inefficient digestions with Xrn1 on *Streptomyces coelicolor* RNA [9]. The authors speculated that the high prevalence of stable secondary structures may account for the lack of degradation of the processed transcripts. Secondary structure and double-stranded recessed 5’ ends of processed transcripts may account for the enzyme’s performance [8] and lead to the identification of spurious TSS. Cappable-seq on the other hand is based on directly targeting TSS and is not confounded by processed ends. Thus, Cappable-seq shows superior performance over dRNA-seq and consequently any TEX based technology.

**Note C**

**Clustering of *E. coli* TSS** :

A closer look at the data at one base resolution identifies secondary starting sites in the immediate surrounding of what appear to be the major primary TSS. Those secondary sites tend to have lower expression relative to the primary sites and are likely to have been initiated from the same promoter. Thus, we clustered TSS sites and retain the position with the highest read score as the TSS. Ideally, the procedure should cluster most of the sites originating from the same promoter while minimizing the clustering of TSS generated from different promoters. To this end, we calculated the number of clusters obtained at distances ranging from 0 to 100 bp. We perform the same analysis with randomly generated positions and estimated that a 5 base cutoff, less than 3% of the independent events and more than 80% of the dependent events are clustered (Supplemental Fig. 9A). We cluster all TSS within a distance of 5 bases into single positions and retain the position with the highest read score and discard secondary positions. We also notice that some TSS do not appear to have secondary starting sites despite being highly expressed. To further examine why some promoters appear to generate multiple TSS positions while others generate a single TSS position, we divided highly expressed TSS (TSS with RRS > 20) into multiple and singlet clustered TSS and examined the sequence specificity. We define singlet clustered TSS as TSS where less than 5% of the relative read score belong to secondary start sites. Conversely we define multiple clustered TSS as TSS where more than 5% of the relative read score belong to secondary start sites. We found that while the promoter specificity (-10) is similar in both groups , about 80% of the singlet clustered TSS have a -1 +1 YR motif characteristic of canonical TSS [12] while less than 50% of the multiple clustered TSS have the YR configuration. Conversely, the promoter specificity (-10) is similar in both groups (Supplemental Fig. 9B).

**Note D**

**Ribosomal TSS can be classified into 3 categories:**

As Cappable-seq eliminates the vast majority of the processed ribosomal RNA, we sought to perform a detailed analysis of the triphosphorylated landscape of transcripts around and overlapping the ribosomal genes. In *E. coli* rrn operons are known to be regulated by two promoters, P1 and P2, which are arranged in tandem and separated by 120 bp. In all 7 rrn operons, we found TSS corresponding to P1 and P2 . Interestingly the ribosomal operons can be classified into 3 groups according to the P1/P2 usage : The first group includes the rrnA, rrnB and rrnC operons and is characterized by a relatively low number of transcripts that initiate at the P1 promoter and a moderate number at the P2 promoter. The second group comprised of the rrnD, rrnE and rrnH operons is characterized by a highly expressed TSS at both the P1 and P2 promoters. The last group corresponds to the rrnG operon and is characterized by an equal number at both P1 and P2 promoters (Supplemental Table 2). Interestingly, we also observe additional positions within the ribosomal operons that are likely TSS. For example we found a candidate sense TSS within the small ribosomal subunit of all operons with a -10 box-like sequence (TACAAT) upstream of the TSS. For most of the ribosomal operons we detect a TSS upstream of the large subunit and a TSS within the large subunit (Supplemental Fig. 8).

**Supplemental Figure Legends**

**Supplemental Fig. 1 : Capping RNA with 3’ DTB-GTP.**

A 25mer T7 triphosphorylated transcript was incubated with VCE in the absence (none) or the presence of 0.5 mM GTP or 0.5 mM 3’DTB-GTP. The reactions were electrophoresed on a 15% TBE (Tris Borate EDTA) Urea polyacrylamide gel. The gel was stained with SYBR® gold.

**Supplemental Fig. 2: 5’ Diphosphate RNA is a substrate for capping with 3’DTB-GTP.**

A 25mer T7 5’ diphosphorylated transcript was in incubated with VCE in the absence(none) or the presence of 0.5 mM GTP or 0.5 mM 2’ DTB-GTP or 0.5 mM 3' DTB-GTP. The reactions were electrophoresed on a 15% TBE Urea polyacrylamide gel and stained with SYBR® gold. The diphosphorylated transcript had been prepared from the 25mer triphosphate transcript by incubation with Saccharomyces cerevisiae Cet1p [13]. As a demonstration of the conversion by Cet1p to the diphosphate the transcript was capped with Saccharomyces cerevisiae CEG, whereas the 25mer triphosphate was not a substrate for Saccharomyces cerevisiae CEG (data not shown).

**Supplemental Fig. 3: Capture of 3’ DTB-GTP capped T7 RNA transcript with Streptavidin.**

32P uniformly labeled 300-mer T7 transcript was incubated with VCE and either GTP or 3’DTB-GTP or 2’DTB-GTP (See Methods). The transcripts were adsorbed to streptavidin beads washed and eluted with biotin. The percent of the transcript recovered by elution was determined by scintillation counting.

**Supplemental Fig. 4 : VCE discriminates between 5’ monophosphate and 5’ triphosphate RNA.**

A 21-mer synthetic 5' monophosphate RNA (IDT) lanes 1 and 2 or 25mer T7 triphosphorylated transcript lanes 3 and 4 were incubated with 0.5 mM 3’DTB-GTP. Lanes 1 and 3 contained no VCE; lanes 2 and 4 contained VCE. The reactions were electrophoresed on a 15% TBE Urea polyacrylamide gel and stained with SYBR® gold.

**Supplemental Fig. 5: Decapping DTB-G capped RNA with RppH**

A 3’ DTB-GTP capped 25mer T7 transcript was incubated with 0, 0.1, 0.3 and 1.0 ul of RppH for 30 minutes at 37 degrees in 1X Thermopol Buffer. The reactions were electrophoresed on a 15% TBE Urea polyacrylamide gel and stained with SYBR® gold.

**Supplemental Fig. 6: Decapping DTB-G and 7mG capped RNA with RppH**

A mixture of the 25-mer transcript capped with either 3’ DTB-GTP or GTP were incubated with 0, 0.005, 0.05, and 0.5 ul of RppH for 30 minutes at 37 degrees in 1X Thermopol Buffer. The reactions were electrophoresed on a 15% TBE Urea polyacrylamide gel and stained with SYBR® gold.

**Supplemental Figure 7 : Comparison between Cappable-seq and dRNA-seq.**

A. Histograms showing the distribution of reads (in % of total mapped reads) mapping to intergenic regions (light blue), protein coding regions (purple), tRNA (red) and rRNA (dark red) for the unenriched control library, Cappable-seq library, Xrn1 treated library and Xrn1 minus library. Cappable-seq library show a strong depletion of reads mapping to rRNA compare to control while dRNA-seq show an enrichment of reads mapping to rRNA and tRNA in the Xrn1 treated library. B. Enrichment/depletion of known processed sites in Cappable-seq and dRNA-seq compare to controls, function of the mean of normalized trimmed read counts at these sites. Most of the processed sites are depleted in Cappable-seq while most of the processed sites from tRNA (blue) and some rRNA (orange) are enriched in dRNA-seq. The rRNA processed sites enriched in dRNA-seq correspond to the processed site of the mature 5S RNA. C. Enrichment score for all positions in the genome passing read threshold (RRS > 1.5) in either the assay or control library for both Cappable-seq and dRNA-seq experiments. Negative scores are depleted regions and positive scores are enriched regions in Cappable-seq or dRNA-seq compared to control. Red points are annotated TSS from Regulon DB.

**Supplemental Figure 8 : Enrichment scores across the seven ribosomal operons in E.coli.**

For each position in the seven ribosomal operons the enrichment score is calculated as described in methods. Only enriched positions with a relative read score of greater than 1.5 in Cappable-seq library are candidate TSS (red). Grey boxes correspond to intragenic regions and light-blue boxes correspond to rRNA or tRNA. 26 bona-fide TSS are found within the rRNA genes.

**Supplemental Figure 9 : Clustering of TSS.**

A. Plot of the total number of clusters function of the distance cutoff (in bp) for the real data (black) and randomly generated positions across the *E.coli* genome (blue). At a distance cutoff of 5, the estimated percentage of dependent and independent events are 80% and 3% respectively. B. Sequence logo at promoters of precise and imprecise TSS. The information content at each position is measured in bits. Positions varies from -15 base to +2 up and downstream the TSS (+1). Position weight matrices logo generated using WebLogo [14].

**Supplemental Figure 10 : Characterization of the Cappable-seq specific TSS and Cappable-seq TSS common to the composite dataset of known TSS.**

A. Distribution of enrichment scores for TSS that are overlapping with the composite dataset of known TSS (red) and Cappable-seq specific TSS (green). B. Distribution of RRS for TSS common to the composite dataset of known TSS (red) and Cappable-seq specific TSS (green).

**Supplemental Table 1 : Clustered TSS in E.coli**

Chromosomal positions of the 16359 Cappable-seq TSS (in gtf format). Column 5 corresponds to

the RRS score of the TSS. Last column corresponds to composite information : chr_pos_RRS_orientation_enrichment. (see Additional file 2 )

**Supplemental Table 2 : Ribosomal TSS**

| **Ribosomal operon** | **rrsP1** | **rrsP2** | **Groups** |
| --- | --- | --- | --- |
| rrnA | 255 (4033262) | 5061 (4033379) | 1 |
| rrnB | 289 (4164390) | 5189 (4164507) | 1 |
| rrnC | 284 (3939539) | 6444 (3939656) | 1 |
| rrnD | 22268 (3427069) | 9972 (3426962) | 2 |
| rrnE | 27737 (4205886) | 10714 (4205994) | 2 |
| rrnG | 6734 (2729470) | 6154 (2729354) | 3 |
| rrnH | 28075 (223485) | 9626 (223593) | 2 |

Strength of the rrsP1 and rrsP2 promoter (in number of reads) for all 7 ribosomal operons. In parentheses are the chromosomal positions of the TSS (E.coli K12 assembly U00096.2). The TSS are classified into three groups : the highly expressed TSS (red) the medium expressed TSS (orange)and the low expressed TSS (green). The ribosomal operons can be classified into 3 groups based on the combination of strength of the P1 / P2 promoters.

**References**

1. Martin SA, Moss B: Modification of RNA by mRNA Guanylyltransferase and mRNA(guanine-7)methyltransferase from Vaccinia Virions. *JBC* 1975, 250:9330–9335.

2. Banerjee AK: 5’-Terminal Cap Structure in Eucaryotic Messenger Ribonucleic Acids. *Microbiol Rev* 1980, 44:174–205.

3. Song MG, Bail S, Kiledjian M: Multiple Nudix family proteins possess mRNA decapping activity. *RNA* 2013, 19:390–399.

4. Thomason MK, Bischler T, Eisenbart SK, Förstner KU, Zhang A, Herbig A, Nieselt K, Sharma CM, Storz G: Global Transcriptional Start Site Mapping Using Differential RNA Sequencing Reveals Novel Antisense RNAs in Escherichia coli. *J Bacteriol* 2015, 197:18–28.

5. Evguenieva-Hackenberg E, Klug G: New aspects of RNA processing in prokaryotes. *Curr Opin Microbiol* 2011, 14:587–592.

6. Deutscher MP: Chapter 9 Maturation and Degradation of Ribosomal RNA in Bacteria. In *Molecular Biology of RNA Processing and Decay in Prokaryotes*. *Volume Volume 85*. Edited by Condon C. Academic Press; 2009:369–391.

7. Condon C: Maturation and degradation of RNA in bacteria. *Curr Opin Microbiol* 2007, 10:271–278.

8. Jäger D, Förstner KU, Sharma CM, Santangelo TJ, Reeve JN: Primary transcriptome map of the hyperthermophilic archaeon Thermococcus kodakarensis. *BMC Genomics* 2014, 15:684.

9. Romero DA, Hasan AH, Lin Y, Kime L, Ruiz-Larrabeiti O, Urem M, Bucca G, Mamanova L, Laing EE, van Wezel GP, Smith CP, Kaberdin VR, McDowall KJ: A comparison of key aspects of gene regulation in Streptomyces coelicolor and Escherichia coliusing nucleotide-resolution transcription maps produced in parallel by global and differential RNA sequencing. *Mol Microbiol* 2014, 94:963–987.

10. Zhelyazkova P, Sharma CM, Forstner KU, Liere K, Vogel J, Borner T: The Primary Transcriptome of Barley Chloroplasts: Numerous Noncoding RNAs and the Dominating Role of the Plastid-Encoded RNA Polymerase. *Plant Cell* 2012, 24:123–136.

11. Salgado H, Peralta-Gil M, Gama-Castro S, Santos-Zavaleta A, Muñiz-Rascado L, García-Sotelo JS, Weiss V, Solano-Lira H, Martínez-Flores I, Medina-Rivera A, Salgado-Osorio G, Alquicira-Hernández S, Alquicira-Hernández K, López-Fuentes A, Porrón-Sotelo L, Huerta AM, Bonavides-Martínez C, Balderas-Martínez YI, Pannier L, Olvera M, Labastida A, Jimenez-Jacinto V, Vega-Alvarado L, Del Moral-Chávez V, Hernández-Alvarez A, Morett E, Collado-Vides J: RegulonDB v8.0: omics data sets, evolutionary conservation, regulatory phrases, cross-validated gold standards and more. *Nucleic Acids Res* 2013, 41(Database issue):D203–13.

12. Kim D, Hong JS-J, Qiu Y, Nagarajan H, Seo J-H, Cho B-K, Tsai S-F, Palsson BØ: Comparative Analysis of Regulatory Elements between Escherichia coli and Klebsiella pneumoniae by Genome-Wide Transcription Start Site Profiling. *PLoS Genet* 2012, 8:e1002867.

13. Ho CK, Pei Y, Shuman S: Yeast and Viral RNA 5’ Triphosphatases Comprise a New Nucleoside Triphosphatase Family. *J Biol Chem* 1998, 273:34151–34156.

14. Crooks GE, Hon G, Chandonia J-M, Brenner SE: WebLogo: a sequence logo generator. *Genome Res* 2004, 14:1188–1190.
