# Supplementary figures and images for: A novel enrichment strategy reveals unprecedented number of novel transcription start sites at single base resolution in a model prokaryote and the gut microbiome
Source: BMC Genomics. 2016 Mar 8;17:199. doi: 10.1186/s12864-016-2539-z (PMC4782308; doi:10.1186/s12864-016-2539-z)

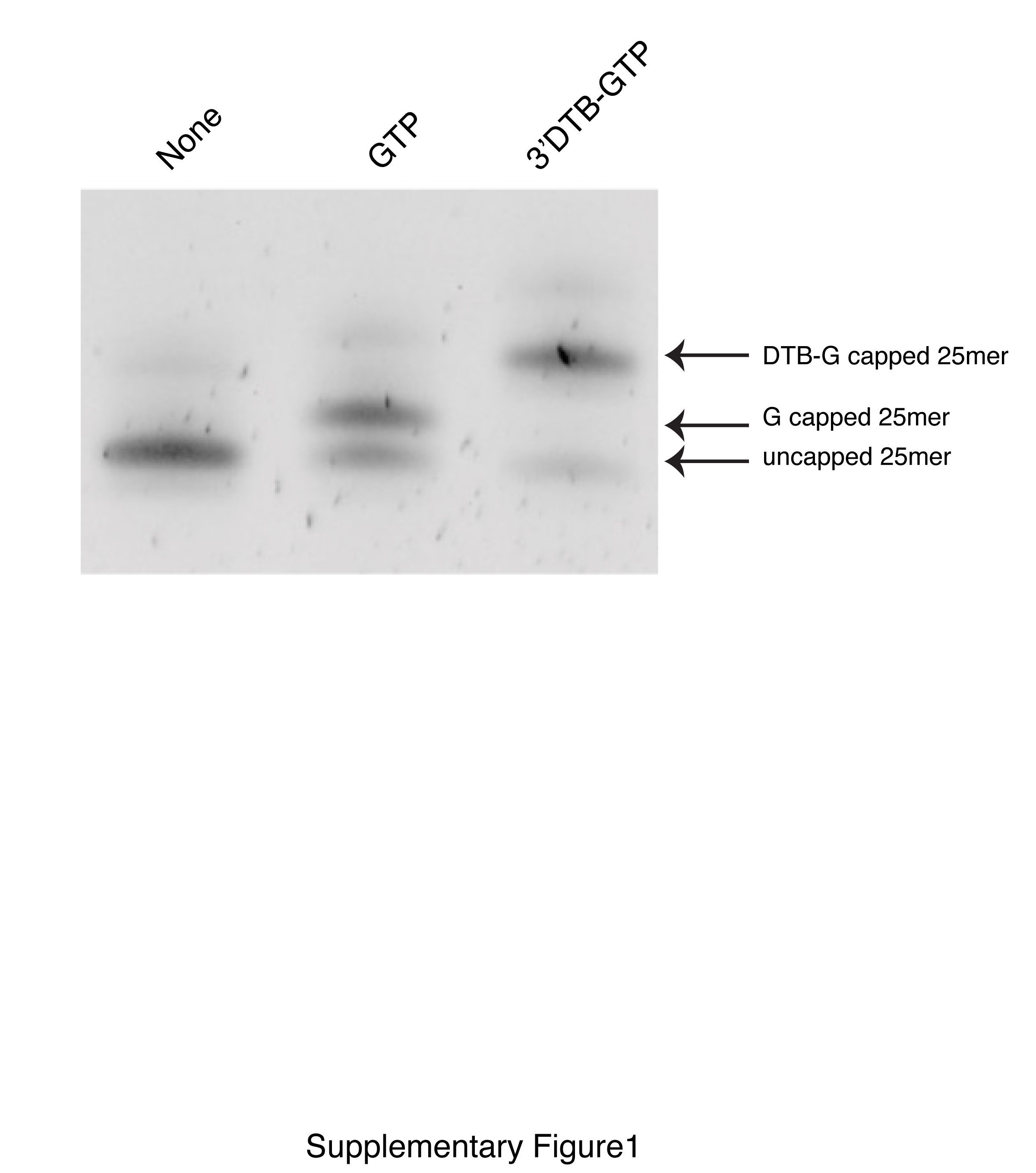

Supplement: Additional file 2: — contains Table S1. (ZIP 9431 kb) [file 12864_2016_2539_MOESM2_ESM.zip › Supplementary_fig1.jpg]

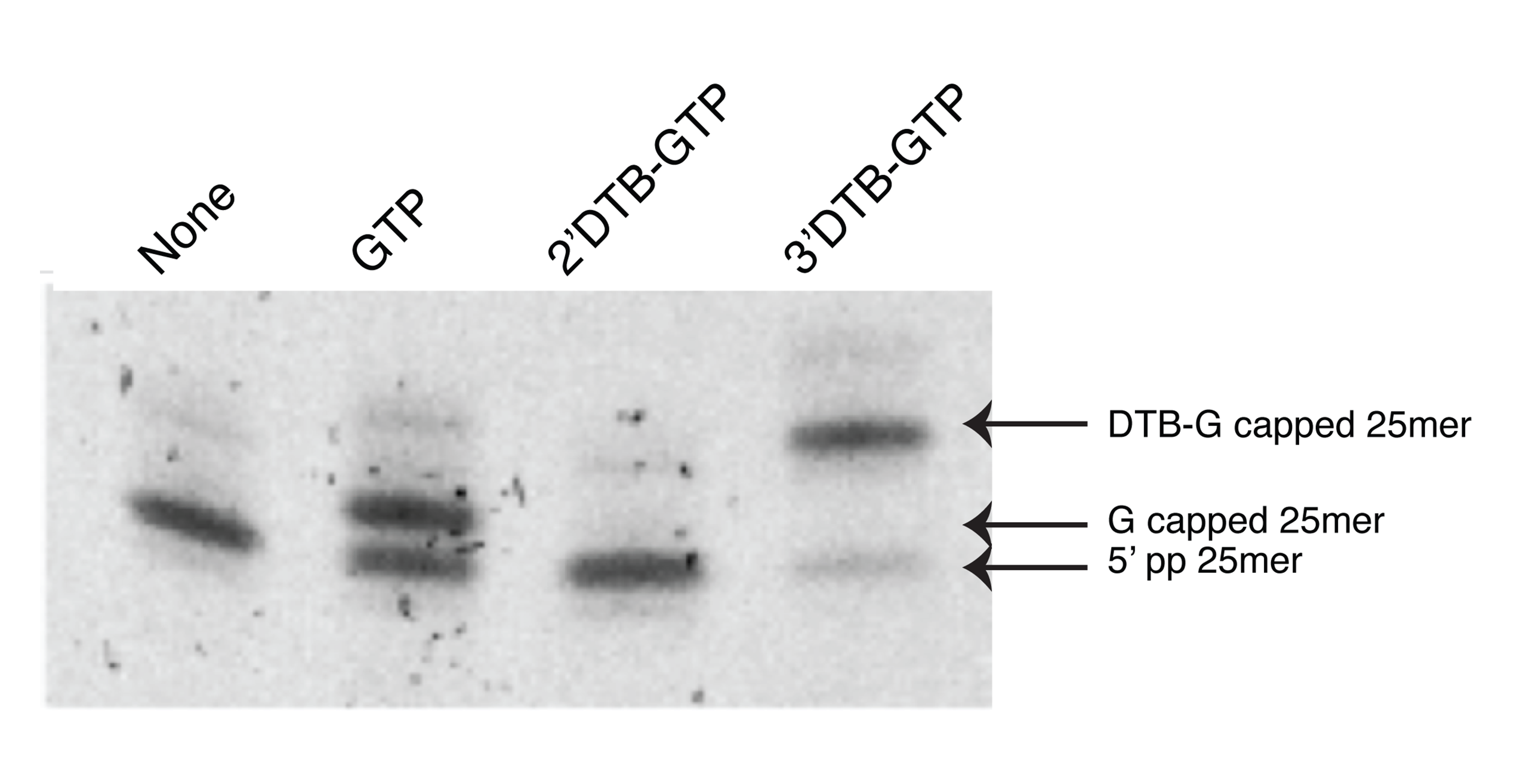

Supplement: Additional file 2: — contains Table S1. (ZIP 9431 kb) [file 12864_2016_2539_MOESM2_ESM.zip › Supplementary_Fig2.tif]

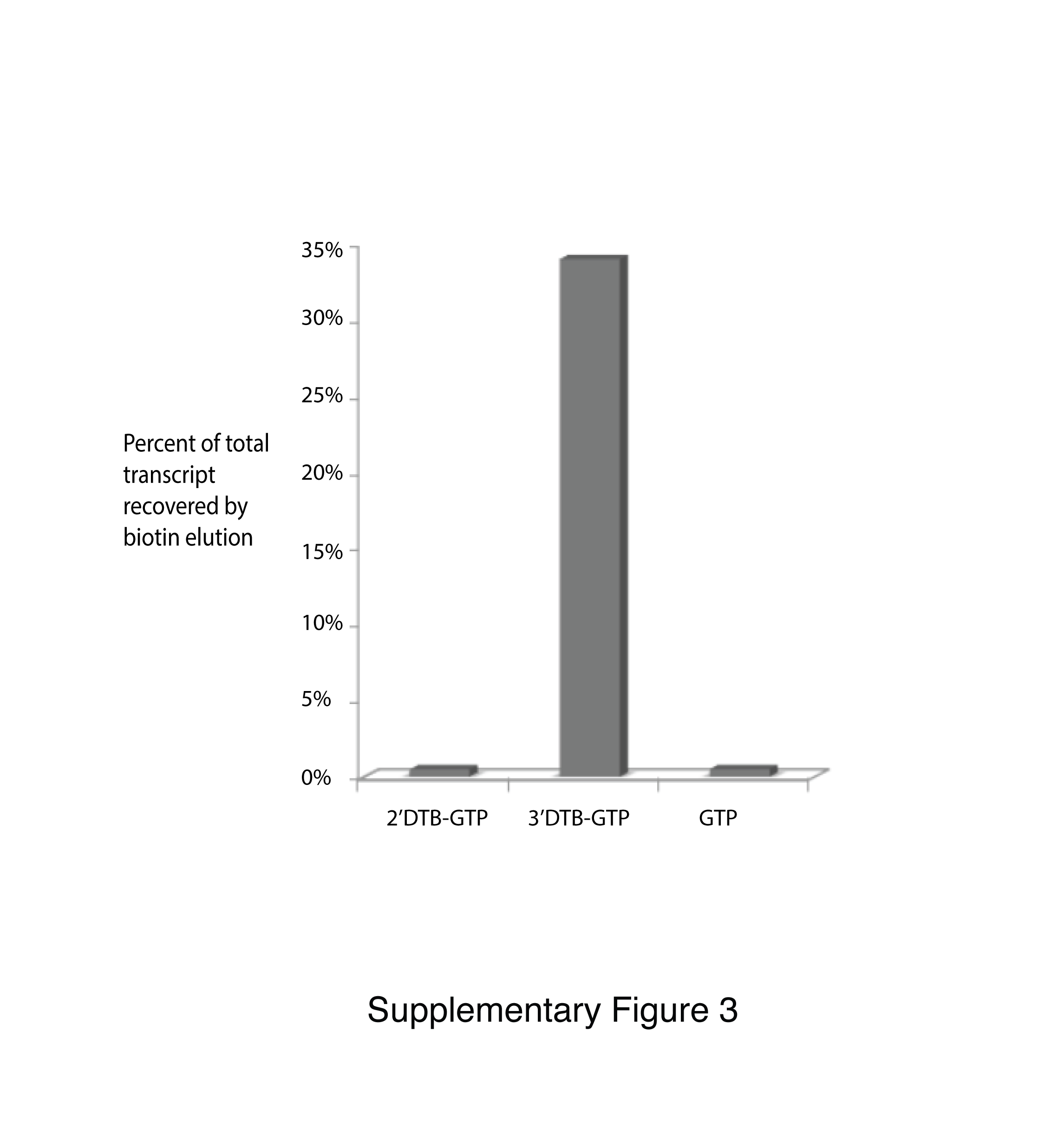

Supplement: Additional file 2: — contains Table S1. (ZIP 9431 kb) [file 12864_2016_2539_MOESM2_ESM.zip › Supplementary_fig3.tif]

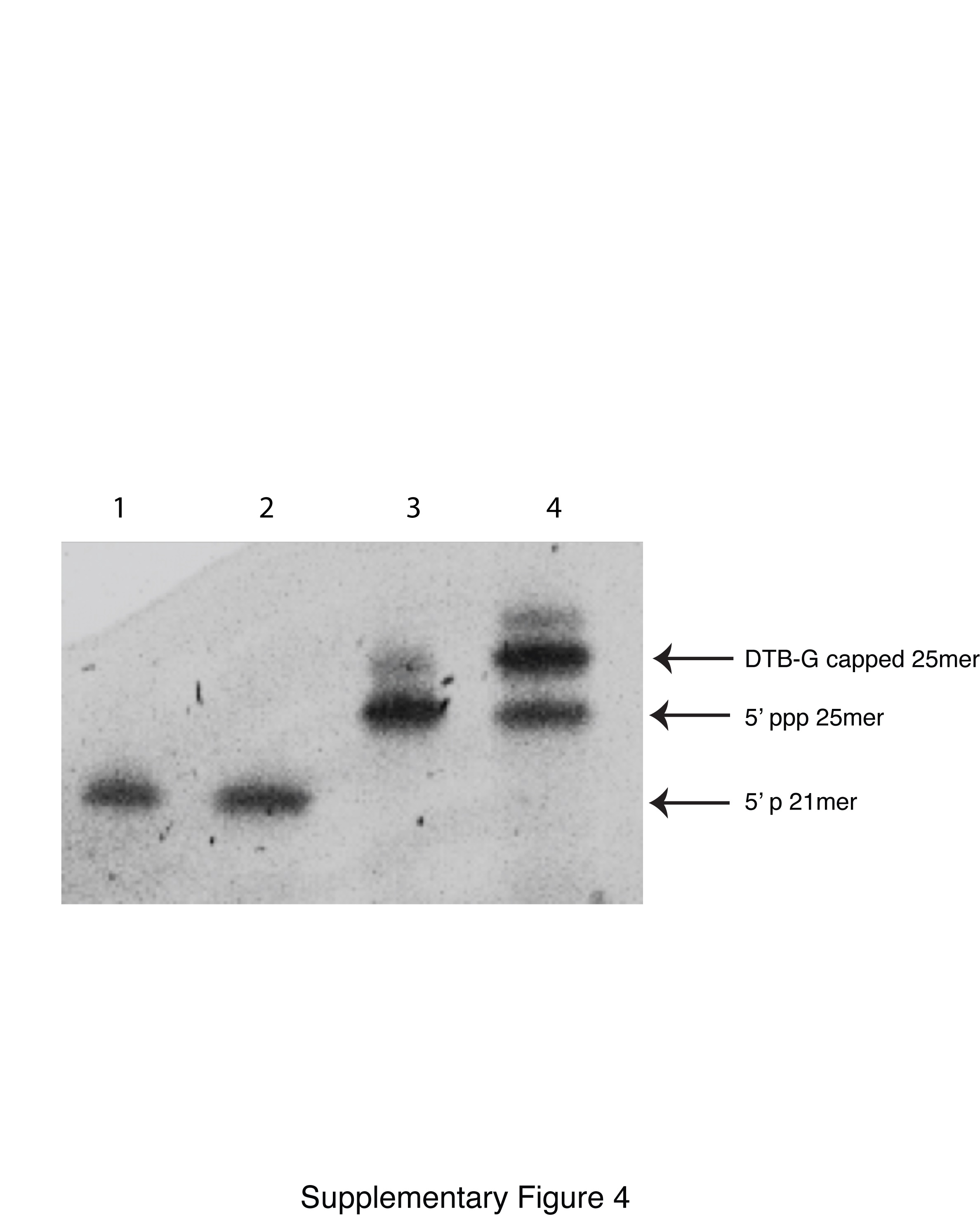

Supplement: Additional file 2: — contains Table S1. (ZIP 9431 kb) [file 12864_2016_2539_MOESM2_ESM.zip › Supplementary_fig4.jpg]

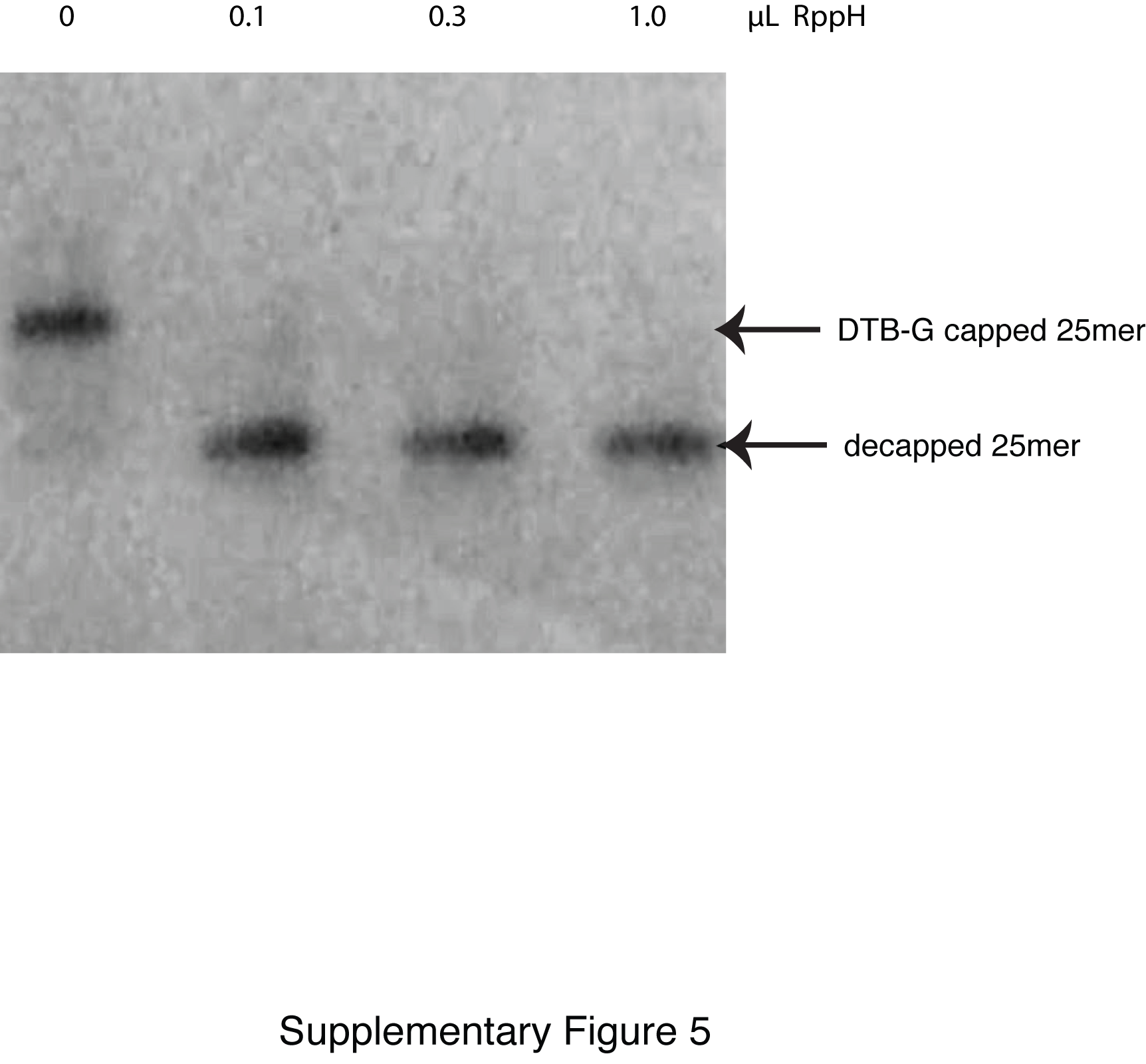

Supplement: Additional file 2: — contains Table S1. (ZIP 9431 kb) [file 12864_2016_2539_MOESM2_ESM.zip › Supplementary_fig5.tif]

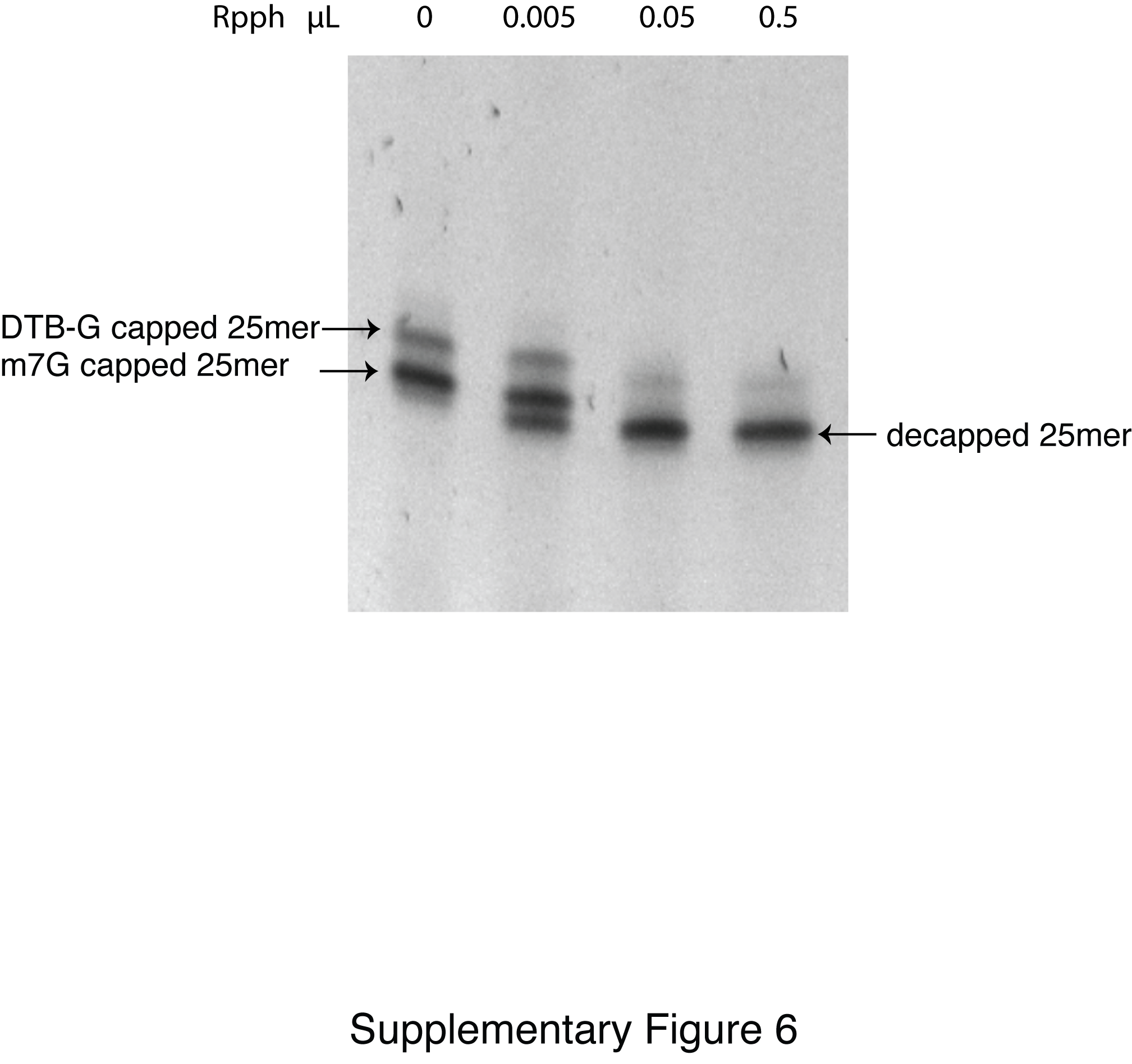

Supplement: Additional file 2: — contains Table S1. (ZIP 9431 kb) [file 12864_2016_2539_MOESM2_ESM.zip › Supplementary_fig6.tif]

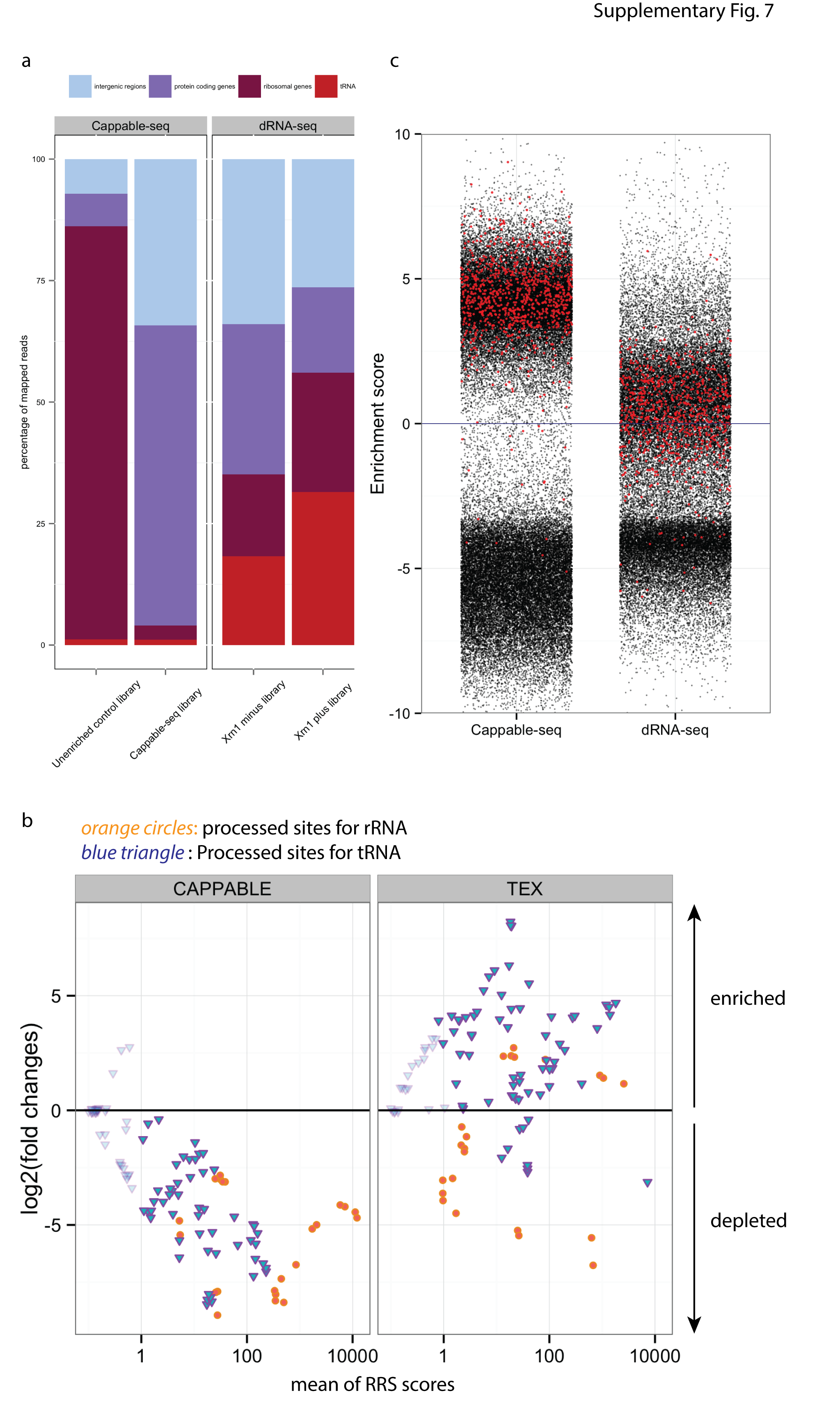

Supplement: Additional file 2: — contains Table S1. (ZIP 9431 kb) [file 12864_2016_2539_MOESM2_ESM.zip › Supplementary_fig7.tif]

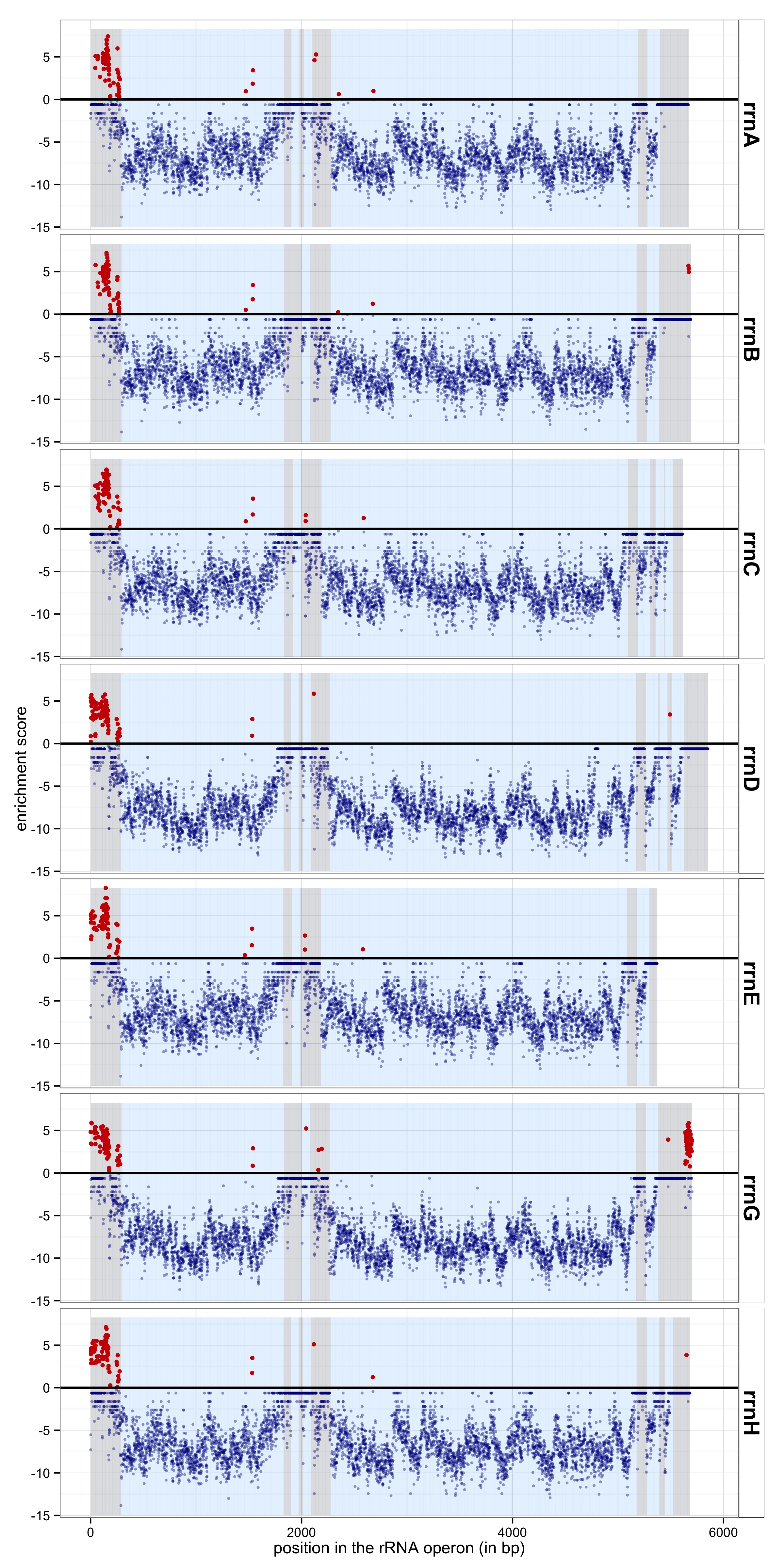

Supplement: Additional file 2: — contains Table S1. (ZIP 9431 kb) [file 12864_2016_2539_MOESM2_ESM.zip › Supplementary_fig8.jpg]

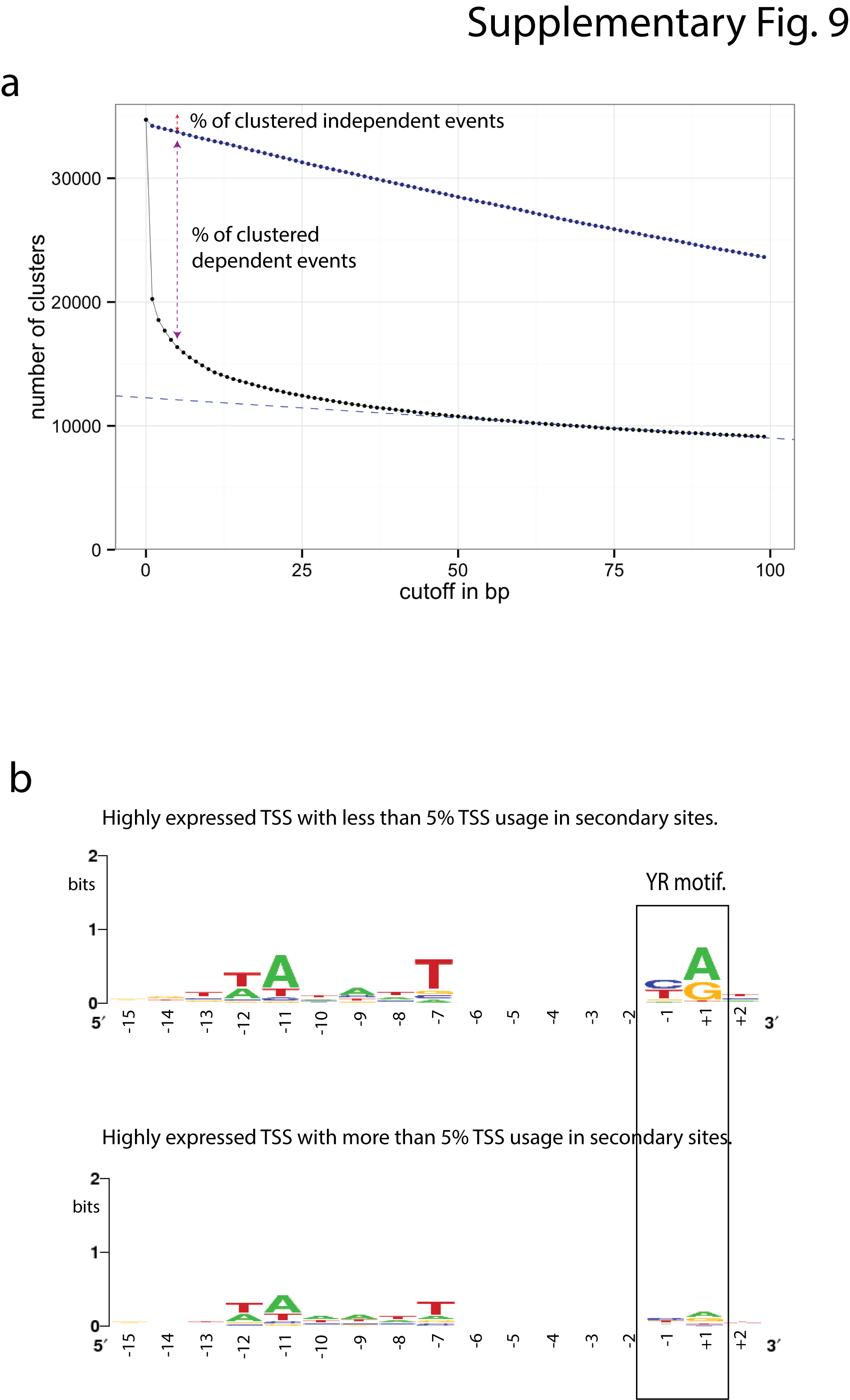

Supplement: Additional file 2: — contains Table S1. (ZIP 9431 kb) [file 12864_2016_2539_MOESM2_ESM.zip › Supplementary_fig9.tif]

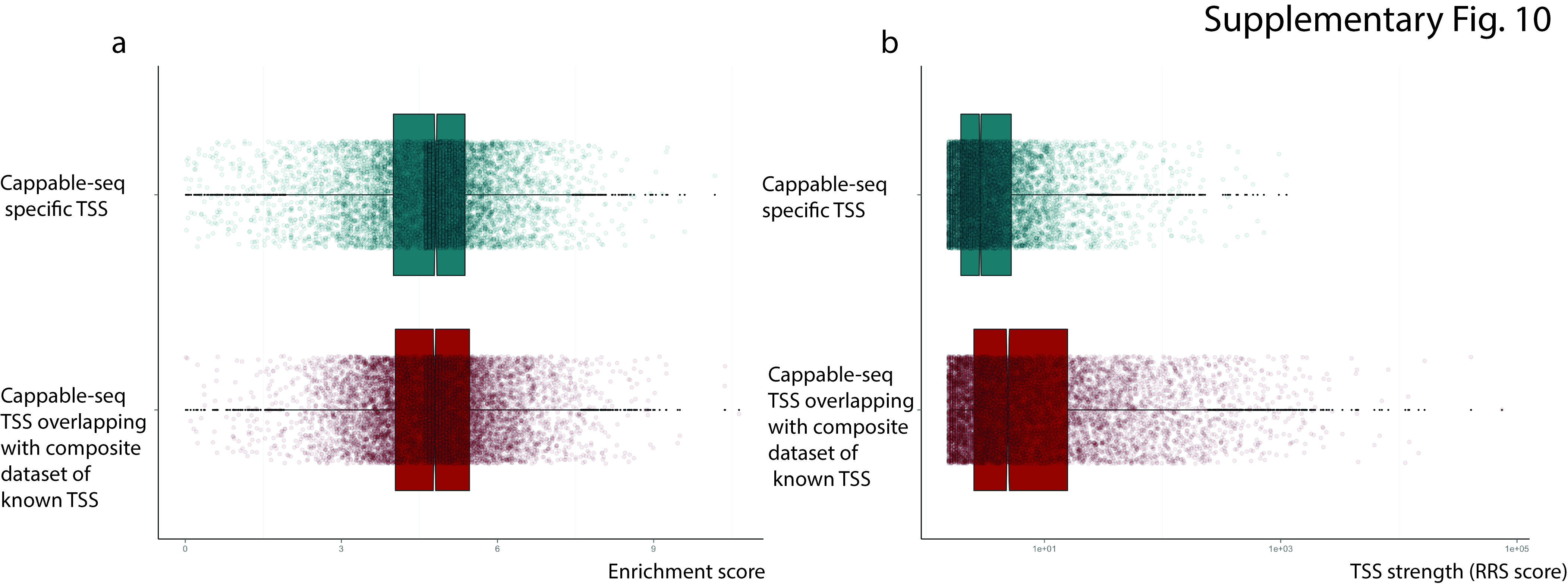

Supplement: Additional file 2: — contains Table S1. (ZIP 9431 kb) [file 12864_2016_2539_MOESM2_ESM.zip › Supplementary_figure10.tif]
